# Drug Burden Index and Its Association With Functional Outcomes in Patients Receiving Hemodialysis

**DOI:** 10.1016/j.xkme.2026.101345

**Published:** 2026-04-01

**Authors:** Cara L. McDermott, Sarah Morton-Oswald, Jonathan Wilson, Juliessa M. Pavon, Cathleen Colón-Emeric, Carl F. Pieper, Rasheeda K. Hall

**Affiliations:** 1 Division of Geriatrics and Palliative Care, Department of Medicine, Duke University, Durham, NC; 2 Department of Population Health Sciences, Duke University, Durham, NC; 3 Geriatrics Research, Education, and Clinical Care, Durham Veterans Affairs Healthcare System, Durham, NC; 4 Department of Biostatistics and Bioinformatics, Duke University, Durham, NC; 5 Division of Nephrology, Department of Medicine, Duke University, Durham, NC

**Keywords:** Activities of daily living, dialysis, functional status, potentially inappropriate medications, renal failure

## Abstract

**Rationale & Objective:**

Psychoactive potentially inappropriate medications (PIMs) are associated with morbidity and mortality in the dialysis population. However, little evidence exists on the association of psychoactive PIMs with functional status, an outcome that matters to patients. Our objective was to examine the association between psychoactive PIM burden and declining functional status.

**Study Design:**

Retrospective cohort analysis.

**Setting & Participants:**

We evaluated a subset of the United States Renal Data System ACTIVE/ADIPOSE study cohort.

**Exposure:**

Drug burden of psychoactive PIMs, measured by annual drug burden index (DBI).

**Outcome(s):**

Gait speed, cognitive function, and dependence in activities of daily living, measured at baseline, 12, and 24 months.

**Analytical Approach:**

We used generalized linear mixed models to evaluate associations between DBI and study outcomes.

**Results:**

Over half (n = 224, 57%) of the 394 patients in our cohort had ≥1 psychoactive PIM prescriptions in the 2-year follow-up period. The most prescribed psychoactive PIMs were gabapentin, zolpidem/eszopiclone, and tramadol. Overall mean (SD) annual DBI at 12 months and 24 months was 1.92 (3.66) and 2.13 (3.89), respectively. We observed a clinically significant 0.07 m/s decline in mean gait speed from 0-12 months, which was not significant in adjusted models. We observed no significant relationship between DBI and cognitive change. Activities of daily living dependence increased from 0-12 months for patients with higher annual DBI (OR, 1.15; 95% CI, 1.01-1.30), but this did not persist from 12-24 months.

**Limitations:**

There are elements of missingness in these retrospective data, which did not impact findings per sensitivity analysis. We used Medicare Part D claims for medication exposure, but we cannot confirm patients took medications as indicated.

**Conclusions:**

Although most patients had ≥1 psychoactive PIM prescription, we did not observe a significant association between annual DBI and declining functional status that persisted over time.

## Plain-Language Summary

Potentially inappropriate medications (PIMs) have harms that can outweigh benefits. Among people receiving dialysis, PIM side effects may be worse because patients are frail and rely on dialysis to eliminate medications. We used the US Renal Data System’s ACTIVE/ADIPOSE study population to see if PIMs are associated with a decrease in people’s cognition, how quickly they walk, and how well they perform activities of daily living (ADLs) such as bathing and dressing. We found that PIMs may reduce walking speed and ADL performance at 1 year, but found no association with cognition, walking speed, or ADL performance at 2 years. This study provides information on the impact of PIMs on function in the general population of patients receiving dialysis.

Adults receiving dialysis have a high symptom burden that negatively impacts their quality of life.^1^ To address their symptoms, clinicians often prescribe psychoactive potentially inappropriate medications (PIMs), defined as medications that may cause more harm than benefit in older adults,^2^ such as muscle relaxants, opioids, gabapentinoids, sedative-hypnotics, and anticholinergics.^3^, ^4^, ^5^, ^6^ However, psychoactive PIMs have been independently associated with morbidity (eg, altered mental status, falls, fractures) and mortality in the dialysis population.^7^, ^8^, ^9^, ^10^, ^11^, ^12^, ^13^, ^14^ For some patients receiving dialysis, the risks of these medications are reasonable if symptoms are controlled and they can maintain their quality of life.^15^ However, psychoactive PIMs may jeopardize quality of life, as prior studies in older adults showed these medications are associated with decline in gait speed, grip strength, and overall physical condition.^16^^,^^17^ Such functional independence matters to patients receiving dialysis, particularly older adults.^18^ Therefore, more evidence is needed to understand if psychoactive PIMs may also potentially worsen physical function and more broadly, quality of life, in adults receiving dialysis.

Because of high symptom burden, including restless legs and chronic pain, some patients receiving dialysis are treated with ≥1 psychoactive PIM. One way to quantify a patient’s total anticholinergic and sedative burden is a drug burden index (DBI), which accounts for dose-response and pharmacologic properties of medications.^19^ Previous studies have found an association between higher DBI and lower physical and cognitive performance in the general population.^19^ Such data for the dialysis population would enhance person-centered prescribing of psychoactive PIMs.

The objective of this study was to identify if higher DBI is associated with declining patient function. We defined function with 3 separate outcomes: gait speed, cognitive function, and dependence in activities of daily living (ADLs). Our hypotheses were that higher annual DBI would be associated with a decline in physical and cognitive function over time, resulting in decreases in gait speed and cognition and an increase in ADL dependence. Because older adults have a higher prevalence of frailty than younger adults receiving dialysis,^20^ we explored if age (grouped as <55 and ≥55 years) modifies these associations.

## Methods

### Study Population

We retrospectively analyzed a subset of the ACTIVE/ADIPOSE study cohort, using 2020 standard analysis files and ACTIVE/ADIPOSE study files from the United States Renal Data System (USRDS) linked to Medicare claims.^21^ The ACTIVE/ADIPOSE study was a prospective, multicenter observational study designed to examine frailty prevalence and progression among patients receiving dialysis. Patients in the ACTIVE/ADIPOSE study were 18 years of age or older, receiving hemodialysis for at least three months and were recruited between 2009 and 2011. Additional information on the original ACTIVE/ADIPOSE study has been published elsewhere.^22^ For this analysis, we required Medicare Part D coverage at study entry to allow for assessment of PIM prescriptions.

### Exposure

The exposure of interest was annual drug burden (ie, DBI)^19^ of psychoactive medications, including but not limited to anticholinergics, benzodiazepines, and opioids. Using medication dose data from Medicare Part D claims, we derived annual DBI for each patient by calculating DBI over 13 distinct time points, specifically, each month including the start and end of the year, prior to follow-up visits. The 12 medication-month segments were used to approximate the annual DBI using the trapezoidal rule.^23^ The terms year 1 and year 2 DBI correspond to medication data from months 0 to 12 and from months 12 to 24, respectively. See Table 2 for a complete list of observed medications/medication classes included in the calculated DBI. Cell sizes <11 must be masked per Medicare data use guidelines; for medications with <11 users at different time points, we report total prescriptions across class (eg, alprazolam and lorazepam categorized together as benzodiazepines).

**Table 2:** Observed Psychoactive Drug Burden Index (DBI) Medications Used by the ACTIVE/ADIPOSE Cohort

DBI Medication	Unique People With 1+ Prescription, n (%)	
	In Year Before 12-mo Follow-up Assessment (N = 393)	In Year Before 24-mo Follow-up Assessment (N = 324)
Gabapentinoids	59 (15.0%)	58 (17.9%)
Zolpidem, eszopiclone	51 (13.0%)	43 (13.3%)
Tramadol	38 (9.7%)	36 (11.1%)
Promethazine	27 (6.9%)	24 (7.4%)
Cyclobenzaprine	21 (5.3%)	26 (8.0%)
Oxycodone	17 (4.3%)	16 (4.9%)
Opioids: fentanyl, hydromorphone, methadone, morphine	30 (7.6%)	16 (4.9%)
Tricyclic antidepressants: amitriptyline, doxepin, nortriptyline	17 (4.3%)	17 (5.2%)
Benzodiazepines: alprazolam, clonazepam, diazepam, lorazepam, temazepam	0	16 (4.9%)
Anticholinergics: cyproheptadine, hydroxyzine, meclizine, scopolamine	17 (4.3%)	16 (4.9%)
Other medications, including baclofen, carisoprodol, chlorpromazine, dicyclomine, methocarbamol, orphenadrine, metaxalone, olanzapine, oxybutynin, paroxetine, perphenazine, solifenacin, tizanidine, tolterodine, trihexyphenidyl, trospium	32 (8.1%)	20 (6.2%)

### Outcomes

The outcomes of gait speed, cognitive function, and ADL dependence were assessed at 3 timepoints (baseline, 12, and 24 months) in the original ACTIVE/ADIPOSE study. Gait speed was defined as the time in meters per second (m/s) to walk 15 feet. Participants walked the distance twice with the fastest attempt recorded as the outcome measurement. A clinically meaningful change in gait speed is defined as ±0.05 m/s, with increasing increments of 0.1 m/s in gait speed predicting greater survival among older adults.^24^ Cognitive function was based on the Kidney Disease Quality of Life Cognitive Function score, which ranges from 0 to 100 with higher scores implying better cognitive function.^25^ ADL dependence was scored by observers from 0 to 4, with the number indicating the need for assistance across 4 domains: bathing, dressing, getting out of a chair, and walking around the home/apartment. Higher ADL dependence scores indicate greater dependence.

### Statistical Analyses

The longitudinal mean trajectories of the 3 outcomes were modeled using generalized linear mixed models to assess for associations with annual drug burden. Models for gait speed and cognitive function assumed a normal distribution and identity link function. The ADL dependence model assumed a negative binomial distribution and log link function. All models included a random subject-specific intercept to account for dependencies within repeated measures from subjects and adjusted for the baseline outcome measure as a covariate. With only 2 follow-up assessments, time was modeled as a discrete binary indicator for year 2, allowing for estimation of model outcomes at 12 and 24 months without need for assumptions on the functional form of time. Annual DBI score was included as a linear continuous term, and a time by DBI interaction was included in all models.

Adjusted models accounted for baseline gait speed, ADLs, and cognition, along with variables previously associated with reduced physical function or frailty among patients receiving hemodialysis: age,^26^ race, Hispanic ethnicity,^27^ sex,^26^ comorbidity burden,^28^ time since initiating dialysis,^29^ use of an assistive device,^30^ dual Medicare-Medicaid eligibility,^31^ total inpatient hospital days in the previous year,^27^ total medication count, and previous year fall history.^32^ Race was included as a confounder due to the effect of racism on patient experience and communication in health care encounters and research study visits by extension.^33^ Medication count was calculated at baseline, 12 months, and 24 months using Medicare Part D claims. Prescribed medication count, including PIMs, was included in all models based on previous evidence of an association between polypharmacy and reduced physical function.^34^ We stratified by age (<55 and ≥55 years) in describing the cohort to view differences in baseline characteristics between older and younger cohort members. We chose 55 as the age cut point given the accelerated physiologic aging seen among patients receiving dialysis and the age distribution of the available cohort.

We observed 2 types of missing data: (1) patients lost to follow-up, (2) and gait speed-specific missingness from patients who indicated that they were unable to complete the timed walk. To address loss to follow-up, we used all available data and generalized linear mixed models using maximum likelihood estimation, which yields unbiased estimates assuming a missing at random mechanism. We performed 2 sensitivity analyses for gait speed. The first sensitivity check imputed a 0 value for those unable to complete the test, while the second assigned a rank, with the lowest rank given to those with an incomplete walk time. We also conducted sensitivity analyses to examine whether controlling for assistive device use or including PIMs in the medication count impacted our results.

This study was reviewed by the Duke University Institutional Review Board (file 00100199) and deemed exempt. All statistical analyses were implemented using SAS/STAT 15.1 software (SAS Institute).

## Results

**Figure 1:**
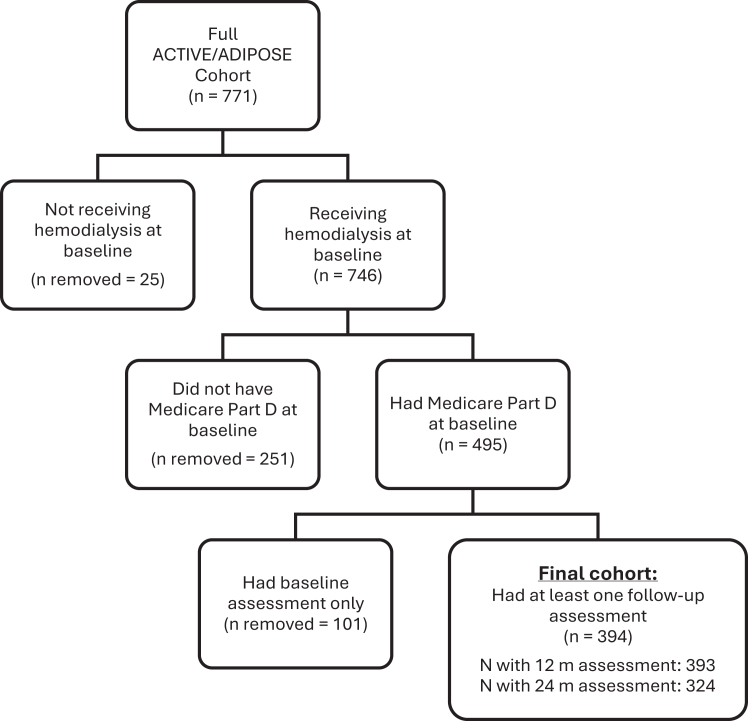
Flow diagram of study cohort creation from the ACTIVE/ADIPOSE cohort.

Of the 771 subjects in the ACTIVE/ADIPOSE study cohort, 746 were receiving hemodialysis at baseline; of those, 495 people had Medicare Part D claims available. Baseline assessment data were missing for 101 people, leaving a final study cohort of 394 people (51% of the ACTIVE/ADIPOSE cohort). Of the 394 participants included in this analysis, 393 (99%) had a 12-month assessment and 324 (82%) had a 24-month assessment (Fig 1).

### Cohort Characteristics

Over half (n = 224, 57%) of the 394 patients had ≥1 prescription for a psychoactive PIM at some point within the 2-year follow-up period, with 124 of those (55%) aged 55 or older (Table 1). A larger percentage of patients aged 55 or younger had such a prescription (63%) compared with those aged 55 or older (53%). Across age groups, we observed a higher mean comorbidity burden, more frequent assistive device use, and higher average number of falls for those with ≥1 psychoactive PIM prescription than those without. Older adults with a PIM prescription were more likely to not have a college education and to be dually enrolled in Medicare and Medicaid versus older adults without a PIM prescription (89% vs 77%; 74% vs 53%, respectively). We provide characteristics of patients of the ACTIVE/ADIPOSE cohort who did not complete a second assessment (24 month follow-up) in supplemental material Table S1.

**Table 1:** Patient Demographics and Clinical Characteristics, Stratified by Age Group and Presence of ≥1 DBI Medication at Baseline

Characteristic	Aged <55, Absence of Rx for a DBI Medication (N = 59)	Aged <55, Presence of Rx for a DBI Medication (N = 100)	Aged ≥55, Absence of Rx for a DBI Medication (N = 111)	Aged ≥55, Presence of Rx for a DBI Medication (N = 124)	Total (N = 394)
**Race**					
Other	12 (20.3%)	21 (21.0%)	46 (41.4%)	39 (31.5%)	118 (29.9%)
Black	47 (79.7%)	79 (79.0%)	65 (58.6%)	85 (68.5%)	276 (70.1%)
**Hispanic ethnicity**					
Yes	<11	<11	13 (11.7%)	14 (11.3%)	37 (9.4%)
No	—	—	98 (88.3%)	110 (88.7%)	357 (90.6%)
**Patient sex**					
Male	45 (76.3%)	61 (61.0%)	60 (54.1%)	55 (44.4%)	221 (56.1%)
Female	14 (23.7%)	39 (39.0%)	51 (45.9%)	69 (55.6%)	173 (43.9%)
**Liu comorbidity index**					
Mean (SD)	1.08 (1.55)	2.02 (2.32)	2.57 (2.84)	3.25 (3.10)	2.42 (2.74)
Median	0.0	1.0	1.0	3.0	1.0
Q1, Q3	0.0, 2.0	0.0, 3.0	0.0, 4.0	0.5, 5.0	0.0, 4.0
Range	(0.0-7.0)	(0.0-8.0)	(0.0-11.0)	(0.0-12.0)	(0.0-12.0)
**Falls in the last year**					
Mean (SD)	0.32 (1.38)	1.20 (5.36)	0.40 (0.86)	0.73 (1.38)	0.69 (2.89)
Median	0.0	0.0	0.0	0.0	0.0
Q1, Q3	0.0, 0.0	0.0, 1.0	0.0, 0.0	0.0, 1.0	0.0, 1.0
Range	(0.0-10.0)	(0.0-52.0)	(0.0-5.0)	(0.0-10.0)	(0.0-52.0)
**Nights in the hospital in the last year**					
Mean (SD)	3.38 (6.79)	5.23 (12.92)	3.99 (9.18)	5.43 (11.40)	4.67 (10.67)
Median	0.0	0.0	0.0	0.0	0.0
Q1, Q3	0.0, 4.0	0.0, 4.0	0.0, 4.0	0.0, 6.0	0.0, 4.0
Range	(0.0-28.0)	(0.0-76.0)	(0.0-59.0)	(0.0-78.0)	(0.0-78.0)
**Assistive device use**					
Yes/missing	<11	<11	40 (36.0%)	57 (46.0%)	116 (29.4%)
No	—	∗^a^	71 (64.0%)	67 (54.0%)	278 (70.6%)
**Education level**					
< College graduate	—	—	85 (76.6%)	110 (88.7%)	331 (84.0%)
≥ College graduate	<11	∗^a^	26 (23.4%)	14 (11.3%)	63 (16.0%)
**Time receiving dialysis**					
Mean (SD)	6.22 (4.86)	7.36 (5.97)	4.17 (4.13)	6.13 (6.24)	5.90 (5.56)
Median	4.5	5.1	2.7	3.9	4.0
Q1, Q3	2.5, 9.2	2.9, 10.8	1.2, 5.9	1.9, 7.9	1.9, 8.5
Range	(0.3-18.8)	(0.1-30.8)	(0.1-22.0)	(0.3-36.6)	(0.1-36.6)
**Dual Medicare-Medicaid eligibility status**					
No	17 (28.8%)	26 (26.0%)	52 (46.8%)	32 (25.8%)	127 (32.2%)
Yes	42 (71.2%)	74 (74.0%)	59 (53.2%)	92 (74.2%)	267 (67.8%)
**Frailty status**					
Yes/missing	14 (23.7%)	23 (23.0%)	41 (36.9%)	57 (46.0%)	135 (34.3%)
No	45 (76.3%)	77 (77.0%)	70 (63.1%)	67 (54.0%)	259 (65.7%)
**Exhaustion**					
Yes/missing	23 (39.0%)	33 (33.0%)	32 (28.8%)	44 (35.5%)	132 (33.5%)
No	36 (61.0%)	67 (67.0%)	79 (71.2%)	80 (64.5%)	262 (66.5%)
**Low physical activity**					
Yes/missing	20 (33.9%)	36 (36.0%)	54 (48.6%)	58 (46.8%)	168 (42.6%)
No	39 (66.1%)	64 (64.0%)	57 (51.4%)	66 (53.2%)	226 (57.4%)
**Slow gait speed**					
Yes/missing	<11	∗^a^	35 (31.5%)	56 (45.2%)	119 (30.2%)
No	—	—	76 (68.5%)	68 (54.8%)	275 (69.8%)
**Weakness**					
Yes/missing	25 (42.4%)	44 (44.0%)	67 (60.4%)	82 (66.1%)	218 (55.3%)
No	34 (57.6%)	56 (56.0%)	44 (39.6%)	42 (33.9%)	176 (44.7%)
**Weight loss**					
Yes/missing	18 (30.5%)	32 (32.0%)	30 (27.0%)	41 (33.1%)	121 (30.7%)
No	41 (69.5%)	68 (68.0%)	81 (73.0%)	83 (66.9%)	273 (69.3%)
**Wheelchair use**					
Yes/missing	<11	<11	13 (11.7%)	16 (12.9%)	36 (9.1%)
No	—	—	98 (88.3%)	108 (87.1%)	358 (90.9%)
**Diabetes**					
Yes/missing/unknown	28 (47.5%)	47 (47.0%)	69 (62.2%)	81 (65.3%)	225 (57.1%)
No	31 (52.5%)	53 (53.0%)	42 (37.8%)	43 (34.7%)	169 (42.9%)
**Weight (kg)**					
Mean (SD)	82.99 (23.94)	83.57 (25.50)	78.65 (17.97)	81.44 (18.60)	81.43 (21.24)
Median	74.3	77.3	77.0	77.2	76.7
Q1, Q3	65.5, 101.4	66.0, 94.8	66.0, 88.6	68.3, 92.3	66.4, 93.2
Range	(43.4-141.0)	(46.5-178.1)	(45.2-130.0)	(51.9-140.5)	(43.4-178.1)
**Height (cm)**					
Mean (SD)	170.66 (9.92)	170.17 (8.98)	166.23 (10.56)	165.89 (9.51)	167.79 (9.95)
Median	171.0	170.0	166.0	165.0	168.0
Q1, Q3	165.0, 178.0	164.0, 176.0	160.0, 173.0	159.0, 172.0	161.0, 175.0
Range	(143.0-188.0)	(151.0-190.0)	(143.0-189.0)	(147.0-193.0)	(143.0-193.0)
**Baseline gait speed (m/s)**					
Mean (SD)	1.06 (0.31)	0.97 (0.23)	0.90 (0.26)	0.79 (0.25)	0.91 (0.27)
Median	1.0	1.0	0.9	0.8	0.9
Q1, Q3	0.9, 1.2	0.8, 1.1	0.7, 1.1	0.6, 1.0	0.7, 1.1
Range	(0.3-2.2)	(0.5-1.8)	(0.3-1.8)	(0.1-1.5)	(0.1-2.2)
**Baseline cognitive function**^b^					
Mean (SD)	91.86 (14.28)	87.21 (16.63)	90.45 (14.29)	86.88 (17.27)	88.72 (15.94)
Median	100.0	96.7	100.0	93.3	100.0
Q1, Q3	86.7, 100.0	80.0, 100.0	86.7, 100.0	80.0, 100.0	80.0, 100.0
Range	(20.0-100.0)	(33.3-100.0)	(33.3-100.0)	(33.3-100.0)	(20.0-100.0)
**Baseline ADL dependence**^c^					
Mean (SD)	0.14 (0.43)	0.20 (0.70)	0.32 (0.85)	0.56 (1.14)	0.34 (0.89)
Median	0.0	0.0	0.0	0.0	0.0
Q1, Q3	0.0, 0.0	0.0, 0.0	0.0, 0.0	0.0, 0.0	0.0, 0.0
Range	(0.0-2.0)	(0.0-4.0)	(0.0-4.0)	(0.0-4.0)	(0.0-4.0)

Note: Data are n (%) unless otherwise specified.

Abbreviations: ADL, activities of daily living; DBI, Drug Burden Index; Q, quartile; Rx, prescription.

a “∗” Symbol indicates values that cannot be disclosed due to adjacent cells that are masked per Medicare data use guidelines (cell size <11).

b Kidney Disease Quality of Life (KDQOL)-Cognitive Function score (higher scores imply better cognitive function).

c ADL dependence rated from 0 to 4 on the need for assistance in bathing, dressing, getting out of a chair, and walking around the home/apartment. Higher scores indicate greater dependence.

### Psychoactive PIM Prescriptions in the Cohort

**Figure 2:**
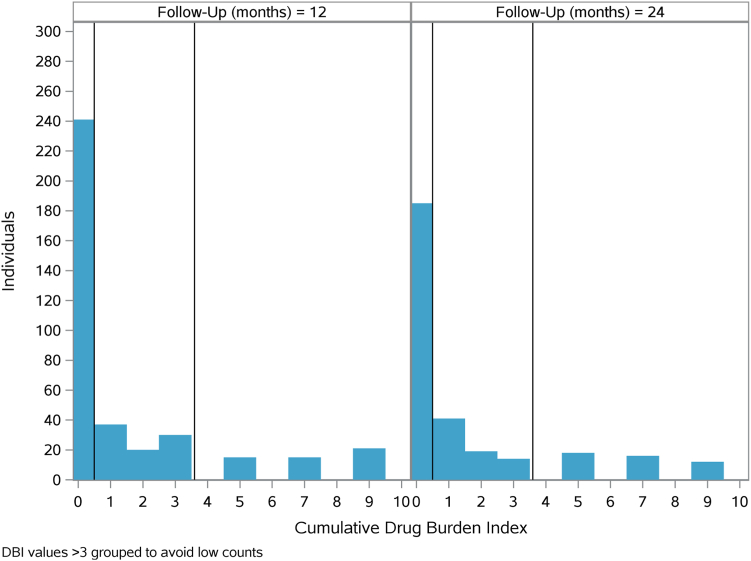
Histogram of Drug Burden Index (DBI) at 12 months and 23 months, with reference lines at 0.1 and 3.15. All bars represent >11 participants.

Of the medications included in the DBI, the most prevalent was gabapentin; 54 (14%) patients received a prescription for gabapentin within a year of their 12-month assessment and 52 (16%) within a year of their 24-month assessment. The next most common medications, in descending order, were zolpidem, tramadol, promethazine, and cyclobenzaprine (Table 2). Among 393 patients at 12 months, the mean annual DBI was 1.92 (standard deviation [SD] 3.66) and ranged from 0 to 23.69, meaning that patients had an average calculated DBI of 1.92, which is considered high exposure to anticholinergic and psychotropic medications.^19^ Annual mean DBI increased to 2.13 (3.89) in the second year of observation among the 324 patients with available data at 24 months, with a similar range (0-22.58). A histogram of DBI scores in the cohort (Fig 2) reveals many patients with DBI of 0 and a right-skewed distribution. For descriptive summaries, we created groupings based on cutoffs at 0.1 and 3.15, creating 3 exposure groups of roughly equal size with annual DBI scores ranging from 0, 0.1-3.15, and 3.16-23.69. Given the skewness of the data and to aid in interpretation, we report associations per increase in annual DBI based on the interquartile range. A 2.3-point difference in DBI is equivalent to the observed sample interquartile range. Stated differently, a 2.3-point difference for a participant is equivalent to their DBI moving from the lower half of the distribution to the upper half.

### Gait Speed

**Figure 3:**
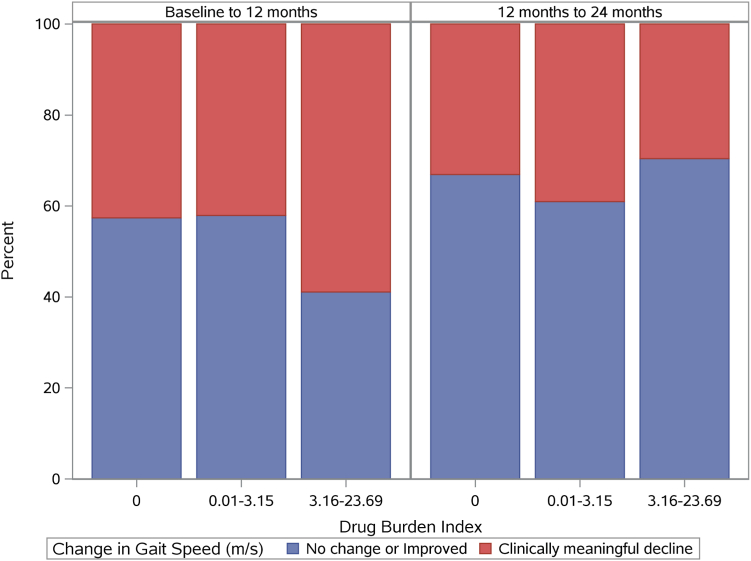
Participants grouped by annual Drug Burden Index and change in gait speed (m/s) over time.

At baseline, 12, and 24 months, 355, 334, and 272 patients had available gait speed data, respectively. The mean gait speed for study subjects decreased by 0.07 m/s from 0 to 12 months, from 0.91 (0.27) m/s at baseline to 0.84 (0.25) m/s at 12 months. Mean gait speed decreased further from 0.84 m/s at 12 months to 0.82 (0.25) m/s at 24 months. Gait speeds ranged from 0.15-2.16 m/s, 0.08-1.70 m/s, and 0.18-1.64 m/s at the 3 respective time points (Table 3). The highest DBI group had the largest proportion of individuals who experienced a decline in gait speed between baseline and 12 months (Fig 3).

**Table 3:** Summary Statistics for the Outcomes of Gait Speed, Cognitive Function and ADL Dependence

Outcome	Follow-up Assessment		
	Baseline	12 mo	24 mo
**Gait speed (m/s)**			
N	355	334	272
Mean (SD)	0.91 (0.27)	0.84 (0.25)	0.82 (0.25)
Range	0.15, 2.16	0.08, 1.70	0.18, 1.64
**Cognitive function**^a^			
N	392	387	319
Mean (SD)	88.72 (15.94)	88.75 (16.77)	87.67 (16.35)
Range	20.00, 100.00	0, 100.00	13.33, 100.00
**ADL dependence**^b^			
N	392	388	319
Mean (SD)	0.33 (0.89)	0.38 (0.95)	0.36 (0.90)
Range	0, 4	0, 4	0, 4

Abbreviation: ADL, activities of daily living; SD, standard deviation.

a Kidney Disease Quality of Life (KDQOL)-Cognitive Function score (higher scores imply better cognitive function).

b ADL dependence rated from 0 to 4 on the need for assistance in bathing, dressing, getting out of a chair, and walking around the home/apartment. Higher scores indicate greater dependence.

Higher year 1 DBI was associated with a slightly lower gait speed of −0.02 m/s at 12 months in unadjusted analyses (95% CI, −0.03 to −0.01). Higher year 1 DBI (per 2.3-point increment) was associated with a decrease of −0.015 m/s in in gait speed (95% CI, −0.03 to −0.003), after adjusting for confounders. There was no association observed between year 2 DBI and the 24-month follow-up gait speed differences (adjusted difference, 0.0003 m/s; 95% CI, −0.012 to 0.013) (Table 4). Additionally, there was no evidence of a significant difference in the strength of the association within age categories (<55 vs ≥55, *P*-interaction = 0.17). In sensitivity analyses, imputing a value of 0 for those unable to complete the gait speed test, assigning ranks based on gait speed, or including assistive device or medication count as a covariate resulted in conclusions consistent with the main findings.

**Table 4:** Unadjusted and Adjusted Effect Estimates for the Association of Year 1 and Year 2 DBI With the Outcomes of Gait Speed, Cognitive Function, and ADL Dependence

Model	Follow-up Assessment	Unadjusted Model	Adjusted Model
		Comparison Estimate (95% CI)	Comparison Estimate (95% CI)
Gait speed^a^	12 mo	−0.02 (−0.03, −0.01)	−0.015 (−0.03, −0.003)
	24 mo	−0.004 (−0.02, 0.01)	0.0003 (−0.012, 0.013)
Cognitive function^a^	12 mo	−1.20 (−2.16, −0.25)	−0.89 (−1.90, 0.12)
	24 mo	−0.95 (−1.92, 0.02)	−0.36 (−1.40, 0.69)
ADL dependence^b^	12 mo	1.15 (1.03, 1.29)	1.15 (1.01, 1.30)
	24 mo	1.04 (0.91, 1.18)	1.05 (0.92, 1.21)

Note: Estimates are per 2.3-point difference in year 1 and year 2 DBIs.

Abbreviations: ADL, activities of daily living; CI, confidence interval; DBI, Drug Burden Index.

a Comparison estimate is of mean differences.

b Comparison estimate is of ratio of average dependencies.

### Cognitive Function

Across the 3 time points of baseline, 12 months, and 24 months, cognitive function data for 392, 387, and 319 patients, respectively, was available for analysis. The mean cognitive function scores were 88.72 (15.94), 88.75 (16.77), and 87.67 (16.35), ranging from 20-100, 0-100, and 13.33-100, respectively (Table 3). There were no substantial changes over time between groups.

We did not observe an association between year 1 or 2 DBI and change in cognitive scores at 12 months and 24 months, respectively, after adjusting for potential confounders. Year 1 DBI was not associated with mean difference estimates in cognitive scores (−0.89 points; 95% CI, −1.90 to 0.12 at 12 months). We also observed no association with year 2 DBI and mean difference estimates in cognitive scores (−0.36 points; 95% CI, −1.40 to 0.69) (Table 4). There was no evidence of differences in the association by age category (<55 vs ≥55, *P*-interaction = 0.46).

### ADL Dependence

For ADL dependence, 392, 388, and 319 patients had available data for analysis at baseline, 12 months, and 24 months, respectively. The mean ADL dependence at the 3 time points was 0.33 (0.89), 0.38 (0.95), and 0.36 (0.90), ranging from 0 to 4 (Table 3). We found no substantial difference in outcomes over time.

We observed that individuals with higher year 1 drug burden had additional ADL dependencies at 12 months. Individuals with a 2.3-point higher year 1 DBI had a 1.15-fold (95% CI, 1.01-1.30) increase in ADL dependency at 12 months after adjustment for confounders, compared with those with lower year 1 DBIs. There was no association between year 2 DBI scores and ADL dependencies (1.05; 95% CI, 0.92-1.21) (Table 4). The risk of additional dependencies was not significantly altered by age category (<55 vs ≥55, *P*-interaction = 0.95).

## Discussion

We aimed to determine if a higher annual drug burden would be associated with declining function in a dialysis cohort unselected for age. Although we expected an association between drug burden and functional status, we only observed association between annual DBI and 2 measures of functional status, gait speed and ADL dependencies, in the first 12 months of follow-up. Of note, this cohort was derived from participants enrolled in a prospective study who had below-average comorbidity burden and the ability to participate in a prospective, observational study. The small decrement in patient function observed suggests a larger decrement may be present in the average dialysis patient (with more comorbid conditions and likely more frailty). Overall, this study informs clinicians on the impact of PIMs on physical function and quality of life, in general, in patients receiving dialysis.

In cohort studies of older adults, DBI has been linked to reductions in gait speed,^16^^,^^35^ cognition,^36^^,^^37^ and independence in ADLs,^38^ as well as increased mortality. Although we did not have similar findings, likely because we had a cohort of patients who selected to enroll in a prospective study, we do demonstrate DBI is a feasible measurement to use in secondary data analysis of the dialysis population. Overall, the mean DBI in this study was >1, which is considered high exposure to anticholinergic and sedative medications^39^ and aligns with other studies that have found significant psychotropic PIM exposure in patients receiving dialysis. DBI is more complex than the simple medication counts often used to define polypharmacy, as it incorporates a patient’s exposure to the medication plus medication characteristics, with higher DBI associated with negative patient outcomes. Additionally, given its focus on anticholinergic and sedative medications that are most likely to cause harm, DBI could be a helpful tool for nephrology clinicians to focus medication reviews on addressing medications with the highest associated risk.

Although we saw a significant association between annual DBI and both gait speed and ADLs in the first 12 months, our findings did not persist over time. This may be the result of multiple factors. First, patients who experience adverse effects from medication typically do so within the first few months of use, which may lead to medication discontinuation; thus, those who would have experienced adverse events were not taking the medication at 12 months. Patients who continue to use PIMs may have better tolerance to medication side effects, resulting in reduced impact on patient outcomes. Second, we analyzed data from the prospective ACTIVE/ADIPOSE study cohort nested within USRDS. The patients enrolled in this study had an average comorbidity burden index ranging from 2-6 points lower than other USRDS studies,^4^^,^^40^, ^41^, ^42^, ^43^ indicating that this is a much healthier group of patients compared to others represented in USRDS data. They may be less likely to receive psychoactive PIMs because they do not have as many illnesses for which these medications are typically prescribed. Additionally, as healthier people, they have more physiologic reserve,^44^^,^^45^ which blunts the potential adverse events of psychoactive PIMs that reduce function, such as falls or altered mental status. Third, we compared measurements across 3 time points: baseline, 12 months, and 24 months. These cross-sectional measurements may have missed fluctuations in participant health; for example, study subjects may have experienced varying cognition, gait speed, and ADL dependence in the time between measurements that are not reflected in the collected data.

Additional research is needed to build on this study’s findings. Recruitment of patients with more comorbid conditions and frailty, plus more frequent assessment of medication use and physical function, would help create a sample more representative of the typical dialysis population whose function may fluctuate frequently over time. Collecting measurements prospectively of whether medications were taken and the context for such use (eg, as needed for pruritis), rather than relying on claims data and patient recall, will help better measure relationships between medication exposure and functional outcomes. After more robust data on the association of psychoactive PIMs and function is collected, future studies to enhance incorporation of patient preferences and medication risks in shared decision-making conversations would be warranted.

We observed a high prevalence of psychoactive PIM prescriptions in this patient cohort, reflected by the average DBI being >1. Although the use of these medications is intended to address side effects of dialysis or treat underlying comorbid conditions, the medications can promote decline and frailty in this patient population. Because of frailty, patients exposed to multiple PIMs are at higher risk of falls and altered mental status, which is why guidelines such as the American Geriatrics Society’s Beers criteria recommend avoiding concomitant PIM use.^2^ If a dialysis patient is already taking a PIM, clinicians should consider PIM alternatives such as massage, physical therapy, cognitive behavioral therapy, and sleep hygiene rather than starting an additional PIM. If PIM use cannot be avoided, then minimizing PIM doses or exposure durations may help reduce risk of adverse events.

The major strength of this study is the unique dataset that included functional outcomes in a dialysis cohort that could be linked to actual prescription claims. However, there are limitations of this study. There are elements of missingness in the data, as some were collected retrospectively. Although we performed sensitivity analyses that note the missing data do not impact on our results, there may be additional unmeasured confounders that are not accounted for. We relied on Medicare Part D prescription claims to determine medication exposure. Claims indicate that the medication was filled, but we cannot confirm that the patients took the medication as indicated. Our study cohort was mostly below the age of 75 when significant cognitive and functional impairment would be expected, thus we may have been underpowered to determine a clinically meaningful change in function. Finally, the cognitive function score used by study investigators, the Kidney Disease Quality of Life Cognitive Function Scale,^25^ has been criticized as having poor sensitivity and moderate specificity for identifying declining executive function and memory.^46^ This may have resulted in some patients with cognitive impairment being misclassified in screening.

We noted substantial use of medications with anticholinergic and sedative properties in adults receiving dialysis in a healthier-than-average cohort. Although the relationship between DBI and functional outcomes was not clinically or statistically significant in adjusted analyses at 24 months, our findings indicate that psychoactive PIM prescribing is common, occurs among multiple drug classes, and may impact ADL dependence in the first year of use. We acknowledge the challenge of managing complex symptoms and comorbid conditions in the dialysis population, where alternatives to PIM use may also not have a robust evidence base. Future research should incorporate patient-reported outcomes across multiple medication classes to generate additional evidence on medication safety and effective symptom management, including nonpharmacologic options, for patients receiving dialysis.
