# Supplementary material for: Drug Burden Index and Its Association With Functional Outcomes in Patients Receiving Hemodialysis
Source: Kidney Med. 2026 Apr 1;8(6):101345. doi: 10.1016/j.xkme.2026.101345 (PMC13202272; doi:10.1016/j.xkme.2026.101345)
Supplement: Supplementary File (PDF) — Table S1 [file mmc1.pdf]

Table S1. Characteristics of patients who did versus who did not complete a second assessment at 24 months for ACTIVE/ADIPOSE, by assessments completed.

Baseline Demographics	Completed 1 Assessment (N=71)	Completed 2 Assessments (N=323)	Total (N=394)
**Race**			
White	14 (19.7%)	52 (16.1%)	66 (16.8%)
Black	42 (59.2%)	234 (72.4%)	276 (70.1%)
Other	15 (21.1%)	37 (11.5%)	52 (13.2%)
**Patient gender**			
Male	40 (56.3%)	181 (56.0%)	221 (56.1%)
Female	31 (43.7%)	142 (44.0%)	173 (43.9%)
**Age Group**			
<55	26 (36.6%)	133 (41.2%)	159 (40.4%)
>=55	45 (63.4%)	190 (58.8%)	235 (59.6%)
**Liu comorbidity index**			
N	71	323	394
Mean (SD)	3.14 (3.27)	2.26 (2.59)	2.42 (2.74)
Median	2.0	1.0	1.0
Q1, Q3	0.0, 5.0	0.0, 4.0	0.0, 4.0
Range	(0.0-12.0)	(0.0-11.0)	(0.0-12.0)
**Falls in the last year**			
N	71	322	393
Mean (SD)	0.49 (1.08)	0.74 (3.16)	0.69 (2.89)
Median	0.0	0.0	0.0
Q1, Q3	0.0, 0.0	0.0, 1.0	0.0, 1.0
Range	(0.0-6.0)	(0.0-52.0)	(0.0-52.0)
**Nights in the hospital in last year**			
N	70	319	389
Mean (SD)	8.80 (16.58)	3.76 (8.64)	4.67 (10.67)
Median	0.5	0.0	0.0
Q1, Q3	0.0, 9.0	0.0, 4.0	0.0, 4.0
Range	(0.0-78.0)	(0.0-76.0)	(0.0-78.0)
**Assistive device use**			
No	46 (64.8%)	232 (71.8%)	278 (70.6%)
Yes	24 (33.8%)	87 (26.9%)	111 (28.2%)

**Education level**			
<college graduate	55 (77.5%)	276 (85.4%)	331 (84.0%)
College graduate/higher	16 (22.5%)	47 (14.6%)	63 (16.0%)
**Time on dialysis**			
N	71	323	394
Mean (SD)	5.62 (5.45)	5.97 (5.58)	5.90 (5.56)
Median	3.9	4.0	4.0
Q1, Q3	1.4, 8.1	1.9, 8.6	1.9, 8.5
Range	(0.1-30.1)	(0.1-36.6)	(0.1-36.6)
**Dual eligibility status**			
No	25 (35.2%)	102 (31.6%)	127 (32.2%)
Yes	46 (64.8%)	221 (68.4%)	267 (67.8%)
**Baseline Gait Speed**			
N	57	298	355
Mean (SD)	0.91 (0.31)	0.91 (0.27)	0.91 (0.27)
Median	0.9	0.9	0.9
Q1, Q3	0.7, 1.1	0.7, 1.1	0.7, 1.1
Range	(0.3-1.8)	(0.1-2.2)	(0.1-2.2)
**Baseline Cognitive Function**			
N	70	322	392
Mean (SD)	86.19 (17.49)	89.28 (15.56)	88.72 (15.94)
Median	93.3	100.0	100.0
Q1, Q3	73.3, 100.0	80.0, 100.0	80.0, 100.0
Range	(33.3-100.0)	(20.0-100.0)	(20.0-100.0)
**Baseline ADL Dependence**			
0	57 (80.3%)	270 (83.6%)	327 (83.0%)
1+	13 (18.3%)	58 (18.0%)	71 (18.0%)

ADL=activities of daily living; Q1=quartile 1; Q3=quartile 3
Some categories/cells collapsed to prevent reporting of <11 subjects per cell.
